# Inhibition of Polycomb Repressive Complex 2 activity reduces trimethylation of H3K27 and affects development in *Arabidopsis* seedlings

**DOI:** 10.1186/s12870-019-2057-7

**Published:** 2019-10-16

**Authors:** Veronica Ruta, Chiara Longo, Alessandra Boccaccini, Valentina Noemi Madia, Francesco Saccoliti, Valeria Tudino, Roberto Di Santo, Riccardo Lorrai, Raffaele Dello Ioio, Sabrina Sabatini, Roberta Costi, Paolo Costantino, Paola Vittorioso

**Affiliations:** 1 Istituto Pasteur Italia-Fondazione Cenci Bolognetti, Sapienza Università di Roma, Piazzale Aldo Moro 5, 00185 Rome, Italy; 2 Dipartimento di Biologia e Biotecnologie “C. Darwin”, Sapienza Università di Roma, Piazzale Aldo Moro 5, 00185 Rome, Italy; 3 Dipartimento di Chimica e Tecnologie del Farmaco, Dipartimento di Eccellenza 2018-2022, Sapienza Università di Roma, Piazzale Aldo Moro 5, 00185 Rome, Italy

**Keywords:** Arabidopsis, PRC2, H3K27me3, EZH2 inhibitor, H3K4me3

## Abstract

**Background:**

Polycomb repressive complex 2 (PRC2) is an epigenetic transcriptional repression system, whose catalytic subunit (ENHANCER OF ZESTE HOMOLOG 2, EZH2 in animals) is responsible for trimethylating histone H3 at lysine 27 (H3K27me3). In mammals, gain-of-function mutations as well as overexpression of EZH2 have been associated with several tumors, therefore making this subunit a suitable target for the development of selective inhibitors. Indeed, highly specific small-molecule inhibitors of EZH2 have been reported. In plants, mutations in some PRC2 components lead to embryonic lethality, but no trial with any inhibitor has ever been reported.

**Results:**

We show here that the 1,5-bis (3-bromo-4-methoxyphenyl)penta-1,4-dien-3-one compound (RDS 3434), previously reported as an EZH2 inhibitor in human leukemia cells, is active on the Arabidopsis catalytic subunit of PRC2, since treatment with the drug reduces the total amount of H3K27me3 in a dose-dependent fashion. Consistently, we show that the expression level of two PRC2 targets is significantly increased following treatment with the RDS 3434 compound. Finally, we show that impairment of H3K27 trimethylation in Arabidopsis seeds and seedlings affects both seed germination and root growth.

**Conclusions:**

Our results provide a useful tool for the plant community in investigating how PRC2 affects transcriptional control in plant development.

## Background

Polycomb group (PcG) proteins are a transcriptional repression system for the epigenetic control of cell, tissue and organ differentiation, contributing to correctly attain a variety of plant developmental programs. PcG proteins are grouped into two complexes: POLYCOMB REPRESSIVE COMPLEX 1 (PRC1) and 2 (PRC2). PRC2 is responsible for the trimethylation of lysine 27 of histone3 (H3K27me3), which is recognized by PRC1 to establish a silent chromatin conformation by monoubiquitination of histone H2A [1]. Furthermore, it has been demonstrated that PRC1 activity can also be required for PRC2 recruitment, and that both complexes can function independently [2–4]. Numerous studies have shown that PRC2 and, in turn, H3K27me3 play crucial roles in cell fate determination during development both in animals and plants [5, 6]. More recently, it has been shown that the two Arabidopsis homologous proteins SHORT LIFE (SHL) and EARLY BOLTING IN SHORT DAYS (EBS), following interaction with EMBRYONIC FLOWER 1 (EMF1), are able to recognize the H3K27me3 mark and trigger the repressive state of PRC2 target loci, thus mediating genome-wide transcriptional silencing [7, 8].

PRC2 consists of four subunits, an histone methyltransferase, a WD40 domain protein, a Zn-finger protein and a nucleosome-remodeling protein, first identified in Drosophila where they are respectively encoded by the genes *ENHANCER OF ZESTE (E(Z)), EXTRA SEX COMBS (ESC), SUPPRESSOR OF ZESTE 12 (SU(Z)12),* and *NUCLEAR REMODELING FACTOR (NURF55*) [9].

In Arabidopsis, there are 12 homologs of the Drosophila PRC2 subunits and, in particular, the histone methyltransferase *EZ* is encoded by three homologs (*CURLY LEAF, MEDEA* and *SWINGER; CLF, MEA* and *SWN*), which share a highly conserved SET domain, responsible of the catalytic activity [10]. Different combinations of the four subunits result in three PRC2-like complexes: the EMBRYONIC FLOWER (EMF), VERNALIZATION (VRN) and FERTILISATION INDEPENDENT SEED (FIS), which function in different developmental processes, although sharing some target genes [11, 12].

In flowering plants, the activity of PRC2 is crucial during endosperm formation as it controls the imprinting of several genes, and mutations in the imprinting machinery lead to embryonic lethality [13]. This has severely hindered studies on the function of PRC2 during seed development. An exception is represented by the genetic strategy used by Bouyer and collaborators, who were able to bypass the female gametophytic defect of the *fertilization independent endosperm* (*fie*) mutant through pollination of heterozygous *fie* mother plants with pollen from a *cdka;1-fie* double heterozygous line. This allowed to generate viable homozygous *fie* mutants, derived from seeds where the endosperm was of uniparental (maternal) origin [14, 15].

In mouse, overexpression of *ENHANCER OF ZESTE HOMOLOG 2* (*EZH2*) and the consequent high level of H3K27me3 are hallmarks of several cancers, making *EZH2* an ideal therapeutic target [16]. The first compound described as inhibitor of EZH2 was the 3-deazaneplanocin A (DZNep), which was shown to reduce H3K27me3 levels through depletion of EZH2 protein level, although with a fairly low specifity [17]. Subsequently, efforts in producing selective inhibitors of EZH2 by means of high-throughput screenings have been highly promising [18–21]. Among the compounds identified, the dual inhibitor of EZH2/EZH1, UNC1999, has been shown to be highly effective in vitro on both wild-type and both gain- and loss-of-function mutant EZH2. UNC1999 was shown to be able to reduce H3K27me3 levels as well as cell proliferation in a large number of cancer cells, without affecting EZH2 protein level [22, 23]. UNC1999 is representative of a family of inhibitors characterized by a 2-pyridone moiety; another class of selective inhibitors of EZH2, characterized by two benzylidene moieties, were generated and subsequently modified to produce a series of more specific compounds [24, 25].

Remarkably, a pharmacological approach has never been tested on plants, although it may represent a good alternative for the study of PRC2 function. Taking advantage of the homology of the PCR2 catalytic subunities of animals and plants, we have assessed the efficacy of 1,5-bis (3-bromo-4-methoxyphenyl)penta-1,4-dien-3-one (RDS 3434) compound, which belongs to the class of inhibitors characterized by two benzylidene moieties. RDS 3434 has been shown to be specifically active only against EZH2, and to be a selective EZH2 inhibitor in human leukemia cells where it induced heavy cell death in a dose-dependent manner, coupled with a reduction of H3K27me3 levels, without affecting EZH2 protein level [24].

The results we present here indicate the efficacy of the RDS 3434 compound as EZH2 inhibitor on Arabidopsis seeds, thus providing a new powerful tool in studying PRC2 action in plants.

## Results

### Treatment of seeds with the RDS 3434 inhibitor reduces H3K27me3 levels in Arabidopsis seedlings

**Fig. 1:**
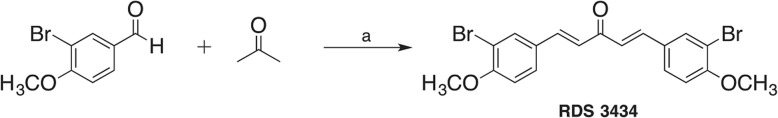
Synthesis and chemical structure of compound RDS 3434. Reagents and conditions: montmorillonite K-10, 100 W, 100 °C, 5 min, 51% yield

The RDS 3434 inhibitor (Fig. 1) has been shown to be specifically active against EZH2 in human leukemia cells, where it induced heavy cell death in a dose-dependent manner [24]. To assess the efficacy of the RDS 3434 inhibitor on Arabidopsis seeds, we grew wild-type seeds on a medium supplied with increasing concentrations of RDS 3434 (30, 60, 120 μM), or with its solvent DMSO (control), for 5 days.

**Fig. 2:**
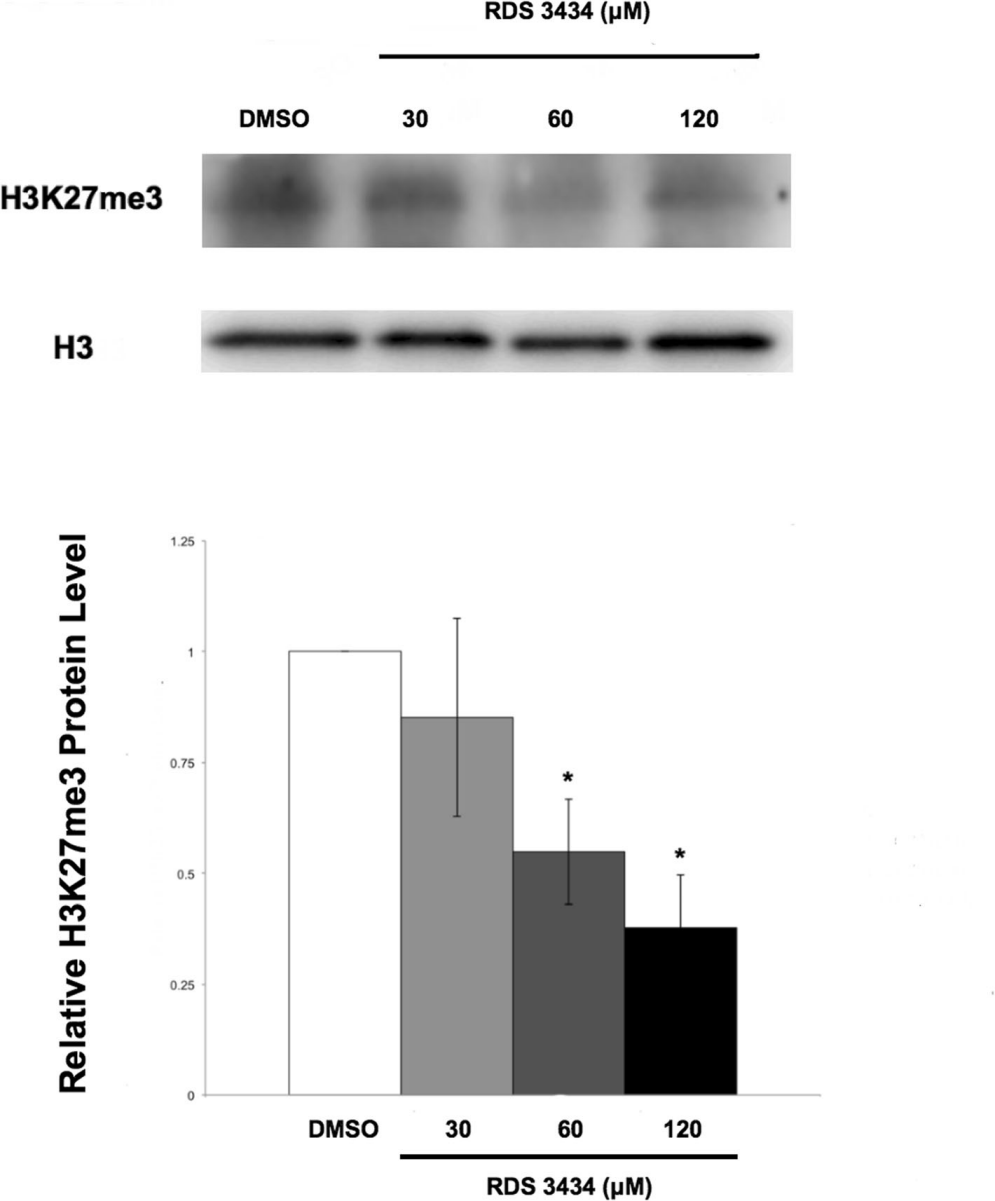
Treatment with RDS 3434 results in a dose-dependent decrease of the total amount of H3K27me3 marked proteins. Immunoblot of 5 days-old wild-type (Ws-4) seedlings directly grown with increasing concentrations (30, 60, 120 μM) of RDS 3434 or DMSO as control. Total proteins were probed with H3K27me3 specific antibodies, and H3 was used as loading control. Western blot (top) and densitometric analysis (bottom). The protein levels are the mean of three biological replicates, presented with SD values. Significant differences were analyzed by *t*-test (*P ≤ 0.05, **P ≤ 0.01)

Immunoblot analysis of total proteins of RDS 3434- or DMSO-treated 5 days-old seedlings was performed with specific antibodies against H3K27me3. Measurement of the amount of proteins marked by H3K27me3 showed that the RDS 3434 inhibitor was effective in a dose-dependent manner: while with 30 μM RDS 3434 the slight decrease (16%) of H3K27me3 marked proteins compared to the control was not significant, at 60 and 120 μM they were reduced by, respectively, 45 and 62% (Fig. 2). CLF is one of the two EZH2 enzymes that play a crucial role during Arabidopsis seedling development, therefore we wondered whether the addition of the inhibitor could further affect H3K27me3 levels in a *clf* mutant. An immunoblot of DMSO- and RDS 3434 (120 μM)-treated *clf-29* mutant seedlings compared to the DMSO- and RDS 3434-treated wild-type (Col) was performed. This analysis revealed that treatment with the inhibitor reduced by 30% the amount of proteins marked by H3K27me3 RDS 3434-treated *clf* seedlings (Additional file 1: Figure S1a), thus corroborating our results. In addition, the ratio of DMSO-treated *clf-29*/WT H3K27me3 protein level confirmed the decrease of H3K27me3 level in *clf-29* compared to the wild-type (Additional file 1: Figure S1b, right).

### The RDS 3434 inhibitor is selectively active against the PRC2 (EZH2) complex

It is known that, in analogy with animal systems, in Arabidopsis the function of the PcG complex is counteracted by the Trithorax Group (TrxG) complex, which catalyzes the trimethylation of lysine 4 of histone 3 (H3K4me3) [26]. We thus verified whether treatment with RDS 3434 caused not only a reduction of the H3K27me3 repressive mark, but also an increase of the H3K4me3 activator mark.

**Fig. 3:**
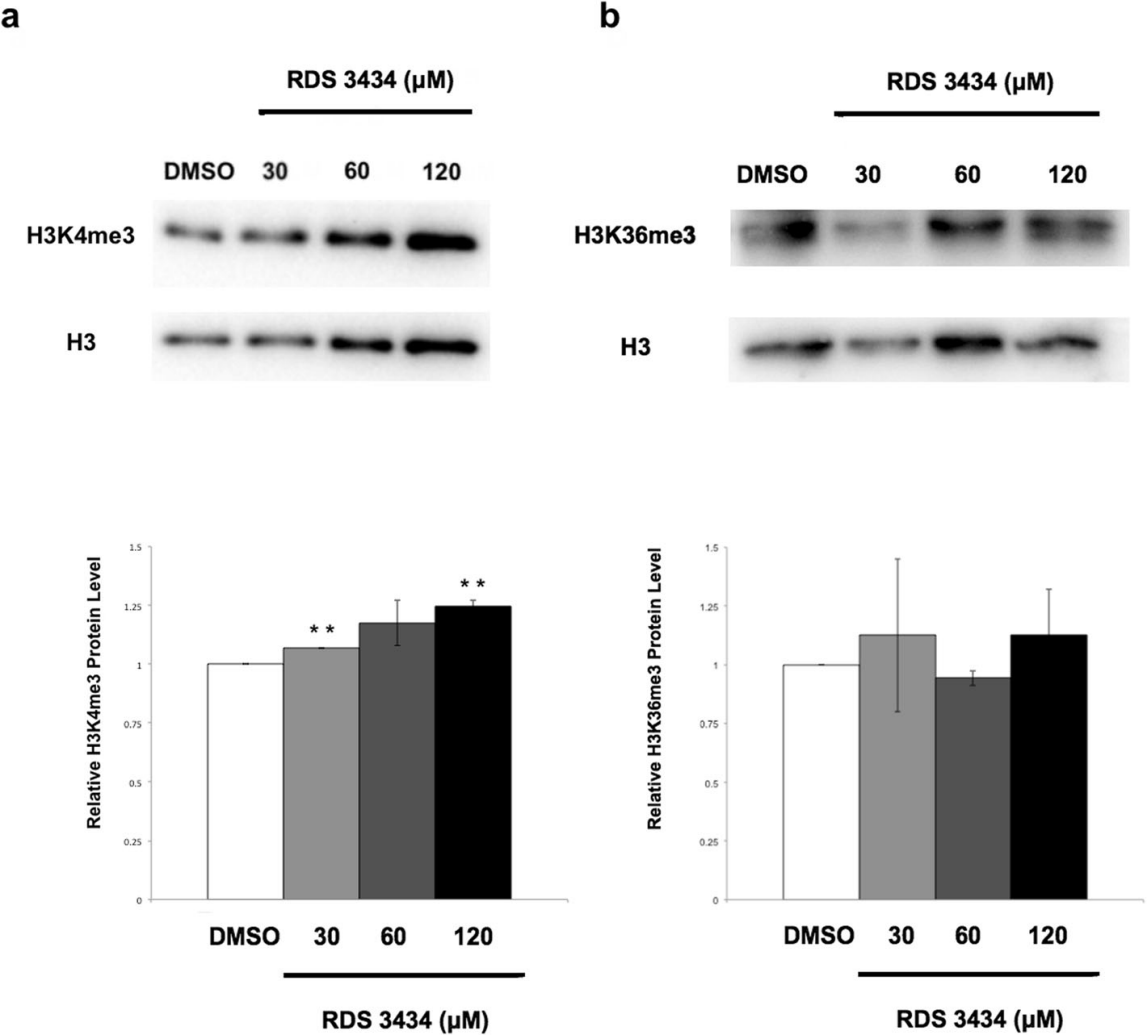
Reduction of the H3K27me3 mark causes an increase of the antagonistic mark H3K4me3. **a**, **b** Immunoblot of 5 days-old wild-type (Ws-4) seedlings directly grown for 5 days in the presence of increasing concentrations (30, 60, 120 μM) of RDS 3434 or DMSO as control. Total proteins were probed with: H3K4me3 (**a**) or H3K36me3 (**b**) specific antibodies. H3 was used as loading control. Western blot (top) and densitometric analysis (bottom). The protein levels are the mean of two biological replicates, presented with SD values. Significant differences were analyzed by *t*-test (*P ≤ 0.05, **P ≤ 0.01)

Immunoblot analysis with specific antibodies against H3K4me3 revealed that treatment with RDS 3434 produced, compared to the control, a small but significant increase in the total amount of H3K4me3 marked proteins at 30 and 120 μM inhibitor (Fig. 3a), consistent with the notion that these antagonistic marks are, in small part, mutually exclusive [27, 28].

To demonstrate that RDS 3434 inhibition was specific for the PRC2 (EZH2) complex over other methyltransferases, we performed an immunoblot analysis with antibodies against H3K36me3, an activating epigenetic mark catalyzed by the SET DOMAIN GROUP 8 (SDG8) [29]. This analysis showed that the H3K36me3 total protein level was not significantly affected even at the highest concentration of RDS 3434 (Fig. 3b), thus suggesting that this inhibitor functions only on the EZH2 metyltransferase.

### Treatment with RDS 3434 increases the expression level of PRC2 target genes

Since PRC2 is a transcriptional repression system, inhibition of EZH2 and consequent decrease of H3K27me3 levels should result in the transcriptional derepression of PRC2 target genes. Thus, we assessed whether treatment with the RDS 3434 inhibitor would actually affect the expression of two indipendent Arabidopsis PRC2 target genes: *DOF AFFECTING GERMINATION 1* (*DAG1*), and *WRKY70*, respectively encoding a Dof and a WRKY transcription factor (TF). *DAG1* encodes a negative regulator of seed germination [30–32], which is marked by H3K27me3 in seeds and seedlings [33] and had been shown to be upregulated in mutant plants lacking PRC2 [14].

*WRKY70* encodes a TF involved in the cross-talk between salicylic acid- and jasmonic acid-dependent defense signaling, and has been reported to be a target of both PRC2 and Trithorax (Trx) [34, 35]. We performed an expression analysis (RT-qPCR) on RNA extracted from RDS 3434-treated (30, 60,120 μM) and DMSO-treated 5 days-old seedlings.

**Fig. 4:**
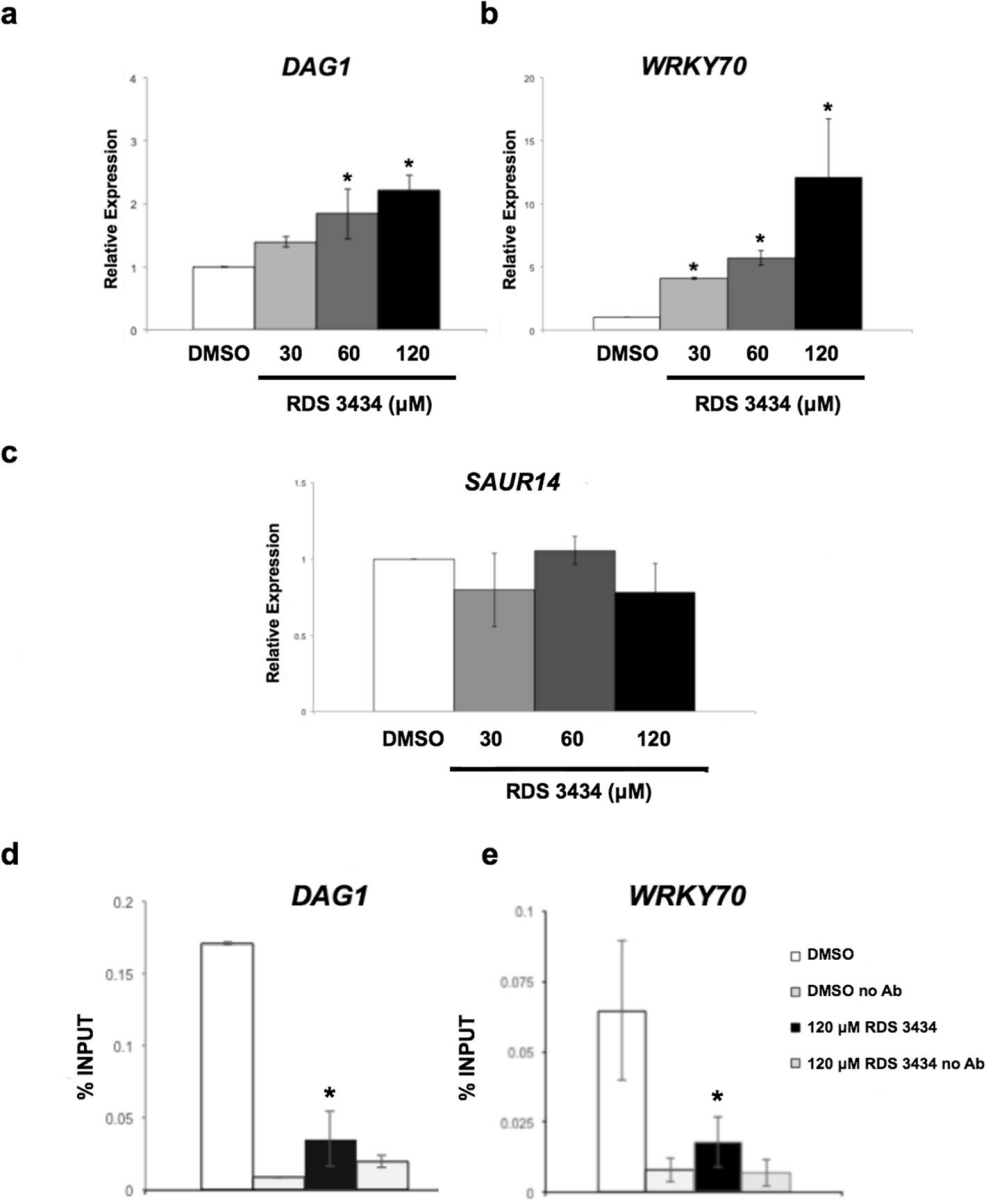
Inhibition of EZH2 results in an increased expression of two PRC2 target genes. **a**, **c** Relative expression level of the PRC2 target genes, *DAG1* (**a**) and *WRKY70* (**b**), and of the non-target gene *SAUR14* (**c**), in wild-type (Ws-4) seedlings directly grown for 5 days in the presence of increasing concentrations (30, 60, 120 μM) of RDS 3434 or DMSO as control. Relative expression levels were normalized with that of the *GAPDHa* (At3g26650) gene, and are presented by the ratio of the corresponding mRNA level in the control, which was set to 1. **d**, **e** Chromatin from samples derived from 5-days old seedlings grown in the presence of 120 μM RDS 3434 or DMSO as control, immunoprecipitated with anti-H3K27me3 antibodies, or without antibodies as a negative control. The amount of DNA for *DAG1* (**d**) or *WRKY70* (**e**) was measured by qPCR. The values of fold enrichment were normalized to input. All the primers used are listed in Table 1. The results were obtained from two independent replicates with SD values. Significant differences were analyzed by *t*-test (*P ≤ 0.05, **P ≤ 0.01)

As shown in Fig. 4a and b, the expression level of both *DAG1* and *WRKY70* significantly increased upon treatment with RDS 3434 in a dose-dependent fashion - respectively 1.9- and 2.3-fold the level of the control for *DAG1* (60 and 120 μM RDS 3434)*,* and 4.2-, 5.8- and 12.1-fold for *WRKY70* (30, 60,120 μM RDS 3434). Under the same experimental conditions, the expression level of *SMALL AUXIN UP RNA 14* (*SAUR14),* which is not a PRC2 target gene [36], was not affected by treatment with RDS 3434, thus confirming the efficacy of this inhibitor only for PRC2 (Fig. 4c). We then assessed whether treatment with the inhibitor would actually result in loss of the H3K27me3 repressive mark in the PRC2 target loci *DAG1* and *WRKY70*. To this end, we performed chromatin immunoprecipitation (ChIP) assays with H3K27me3-specific antibodies, or without antibodies as negative control, on samples derived from RDS 3434 (120 μM)-treated and DMSO-treated 5 days-old seedlings. We measured the enrichment of H3K27me3 by amplification, through quantitative (qPCR), of one region in the body of both *DAG1* and *WRKY70* genes, because the H3K27me3 epigenetic mark is usually restricted to the transcribed regions of target genes [37, 38]. Interestingly, in samples derived from RDS 3434 (120 μM)-treated seedlings, the level of H3K27me3 in the *DAG1* and *WRKY70* genes was significantly decreased (Fig. 4d, e), consistently with their increased expression levels.

### Reduction of the H3K27me3 mark affects seed germination and root development

**Fig. 5:**
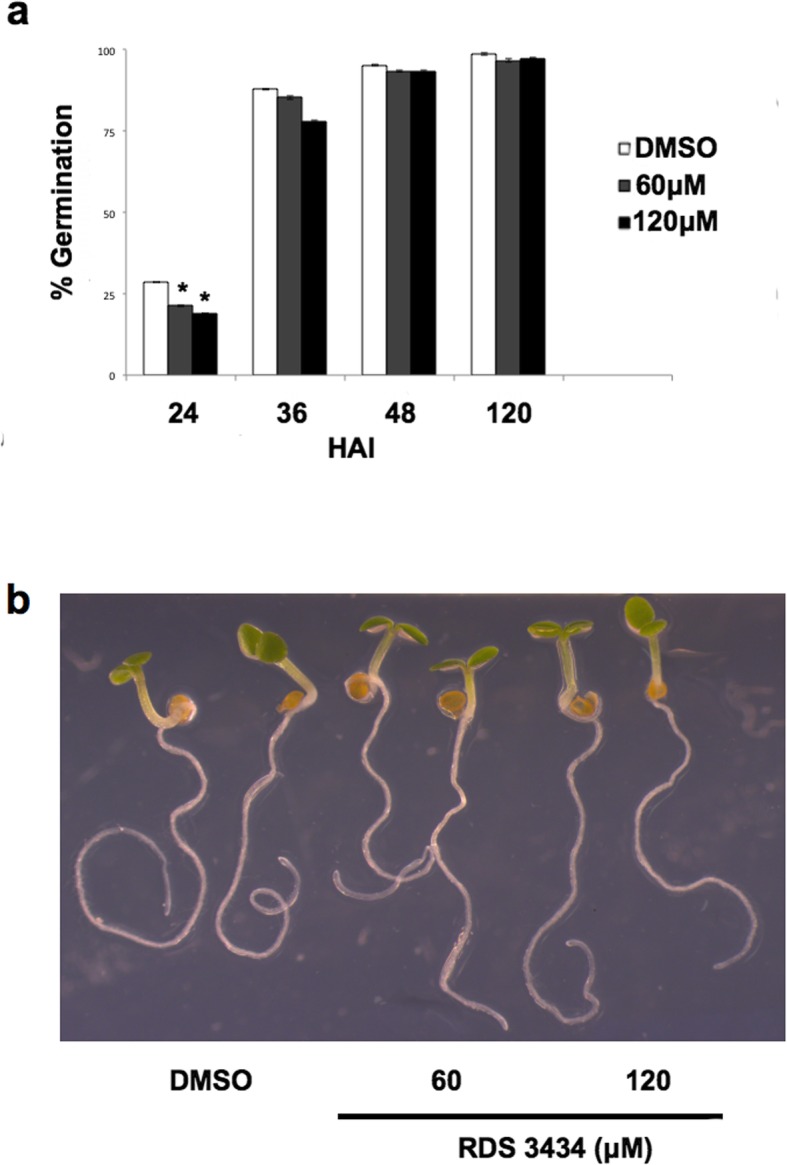
Inhibition of EZH2 results in delayed seed germination. **a** Seed germination assays of wild-type (Ws-4) seeds imbibed in the presence of RDS 3434 (60, 120 μM) or DMSO as control. Germination rate was scored at 24, 36, 48 and 120 HAI (Hours After Imbibition). Data represent the mean of two independent biological replicates each performed in duplicate (25 seeds per replica). Significant differences were analyzed by *t*-test (*P ≤ 0.05, **P ≤ 0.01). **b** 5 days-old wild-type (Ws-4) seedlings directly grown for 5 days in the presence of increasing concentrations (60, 120) of RDS 3434 or DMSO as control

The transcriptional control mediated by PRC2 is crucial during seed germination, as it silences seed specific genes thus allowing proper seedling growth and development [14, 26]. Therefore, we assessed whether treatment with the inhibitor RDS 3434 would affect germination of seeds. As shown in Fig. 5a, treatment with the inhibitor caused a significant reduction of the germination rate at 24 h after imbibition (HAI) - 26.5 and 34.3%, at 60 and 120 μM RDS 3434, respectively.

On the other hand, seedling growth of treated and untreated samples was very similar (Fig. 5b), ruling out the possibility that the observed reduction of H3K27me3 upon treatment with RDS 3434, may pleiotropically affect seedling growth and development. Similarly, 5 days-old seedlings of *clf* or *swn* single mutants do not show severe developmental defects, since only *clf* adult plants are characterized by dwarfism and early flowering, whereas *swn* mutants display very weak phenotypes [39, 40]. Since treatment with RDS 3434 further reduces H3K27me3 level in *clf-29* seedlings, we assessed whether seed germination of mutant seeds would be affected by treatment with RDS 3434 (120 μM). Interestingly, *clf-29* mutant seeds treated with the inhibitor showed a 50% reduction of the germination rate at 24 HAI (Additional file 2: Figure S2a). However, treatment with the inhibitor did not result in more severe phenotypes during seedling development (Additional file 2: Figure S2b).

**Fig. 6:**
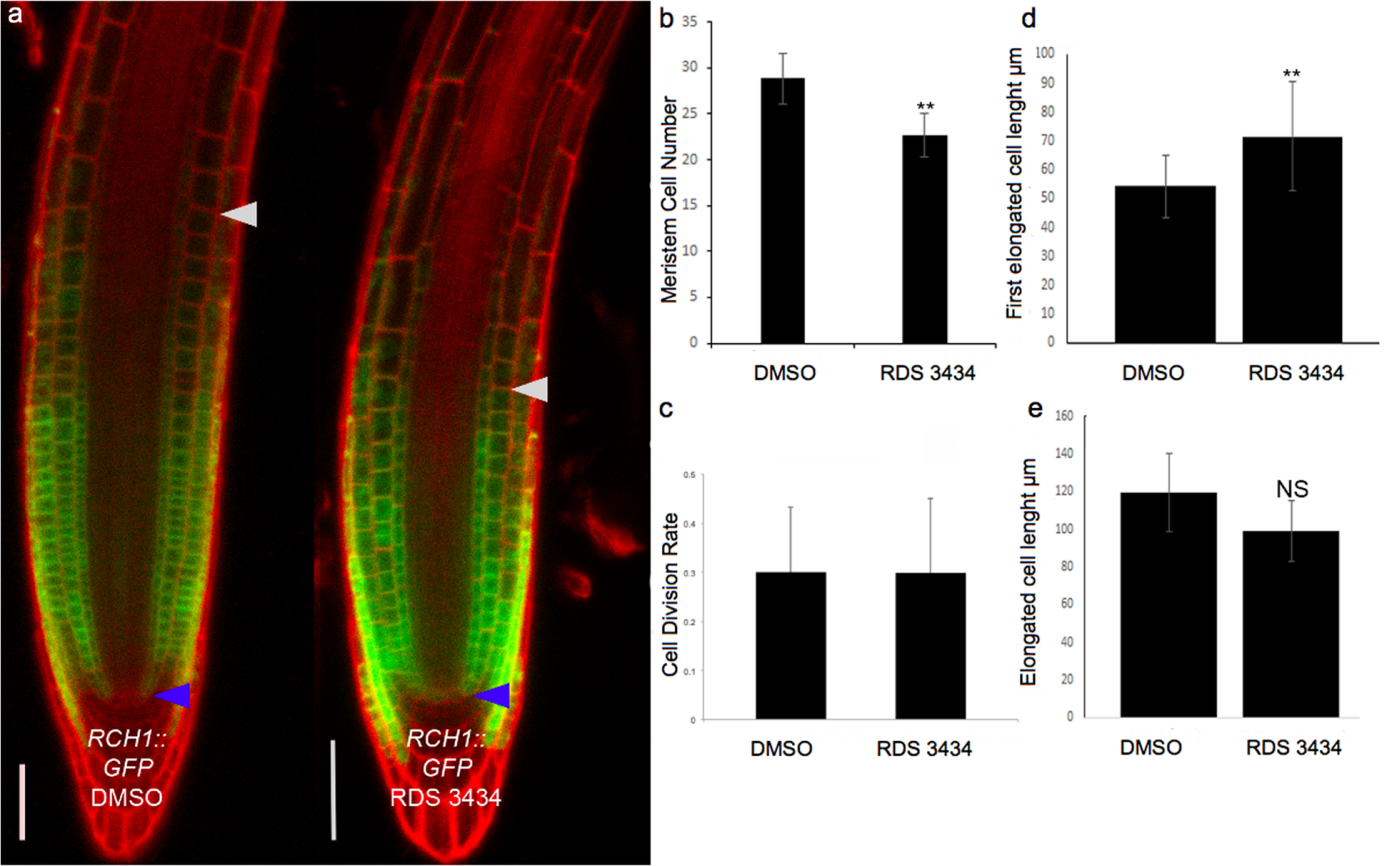
Inhibition of EZH2 affects root development. **a** Confocal microscopy images of *RCH1::GFP* roots from 5 days-old seedlings grown in the presence of RDS 3434 (240 μM) or DMSO as control. Blue and white arrowheads indicate the Quiescent Center (QC) and the cortex Transition Boundary (TB), respectively. **b** Root meristem cell number. **c** Quantification of GUS spots per meristem in treated and untreated of *CYCLINB1;1*_*pro*_*:CDB-GUS* roots. **d**, **e** Length of the first elongated cell (**d**), and of the differentiated cell (**e**) (*n* = 30). Data represent the mean of two independent biological replicates, presented with SD values. Significant differences were analyzed by *t*-test (*P ≤ 0.05, **P ≤ 0.01)

It has been proposed that PRC2 controls primary root growth, since lack of the EZ catalytic subunits CLF and SWN results in short meristem and decreased primary root length [41]. Therefore, we assessed whether treatment with the RDS 3434 inhibitor results in root developmental defects. Although treatment with 60 and 120 μM RDS 3434 inhibitor did not affect root growth (data not shown), a higher dose (240 μM) resulted in a reduced number of root meristematic cells. Consistently, the expression domain of *ROOT CLAVATA HOMOLOG1 (RCH1)*, a gene specifically marking the root meristematic zone [42], is reduced in RDS 3434-treated plants compared to the control, as visualized by a *RCH1-* GFP transcriptional fusion (Fig. 6a, b).

A decrease in meristem size can be caused by a reduced division rate or by a more rapid elongation/differentiation (i.e. exit from the meristem) of meristematic cells. To distinguish between these two possibilities, we first visualized root meristem cells in the G2–M phase in RDS 3434-treated and untreated plants harboring the D-Box *CYCB::GUS* construct, a marker of the G2-M transition [43]: no difference in the cell division potential could be detected (Fig. 6c). To detect possible variation in cell elongation/differentiation, we measured the length of the first elongated and of the fully differentiated cells in both treated and untreated plants. Whereas the length of fully elongated cells was unvaried, the first elongated cells were longer in RDS 3434-treated plants (Fig. 6d, e), indicating that RDS 3434 affects root meristem activities controlling the elongation/differentiation potential. On the other hand, the overall appearance of 240 μM-treated seedling was similar to that of DMSO-treated seedlings (Additional file 3: Figure S3).

## Discussion

In this work, we describe a simple methodology for inhibiting in plants the methyltransferase activity of EZH2, the catalytic subunit of the PRC2 complex, and report the effects of this inhibition on Arabidopsis seed germination and root growth.

PRC2 plays a crucial role in the embryo-to-seedling [14, 44, 45] and vegetative-to-reproductive developmental phase transitions [46, 47]. PRC2 is also essential for endosperm formation, since it controls the parent-of-origin specific expression of a number of genes: lack of the maternal PRC2 function results in derepression of target genes, causing endosperm overproliferation and eventually seed abortion [13, 48]. This has severely hampered studies on the function of PRC2 during seed development.

In mammals, studies on PRC2 function have benefited of the development and use of inhibitors of the catalytic subunit EZH2, among which RDS 3434 whose effectiveness has been proven on the oncogenic human monocyte cell line U937 [24]. Since the catalytic subunit EZH2 is highly conserved between mammals and plants, we tested the effectiveness of RDS 3434 in Arabidopsis and found that indeed it inhibits PRC2-mediated H3K27me3 methylation also in this organism.

Our study does not indicate whether inhibition of methylation is specific for a particular H3K27me3 methyltransferase of the Arabidopsis E(z) group, namely CLF, SWN or MEA, although our data concern the seed-to-seedling transition where CLF and/or SWN are active [11]. Previous genome-wide analyses comparing the global H3K27me3 profile in *clf-28* or *clf-29* mutant seedlings, which revealed a decreased level of H3K27me3 in the mutant lines compared to the wild-type [49–51].

Consistently, our immunoblot analysis showed the decrease of H3K27me3 level in *clf-29* compared to the wild-type, and revealed that treatment with RDS 3434 further reduces the amount of proteins marked by H3K27me3 in the *clf-29* mutant background.

It will be interesting to assess the efficacy of RDS 3434 during gametophyte and endosperm development, where the EZH2 subunit involved is MEA [52].

It has been previously demonstrated that lack of both the catalytic subunits CLF and SWN result in delayed germination: *clf-28 swn-4* double mutant seeds germinate within 4 DAI (Days After Imbibition), while wild-type seeds germinate within 2 DAI [14]. Similarly, seeds of the double mutant *atmib1a atmib1b*, which lacks the E3 ubiquitin ligase subunit BMI of the PRC1 complex [53, 54], reach full germination at 6 DAI whereas the corresponding wild-type seeds germinated at 3 DAI [26]. Consistently, treatment with RDS 3434 affects seed germination in a dose-dependent fashion, as wild-type seeds treated with RDS 3434 showed a significant reduction of the germination rate within the first 36 h compared to untreated seeds; this phenotype is even more pronounced in the *clf-29* mutant background, thus corroborating the effectiveness of RDS 3434 as an inhibitor. It should be pointed up that the *clf-29* single mutant has been previously characterised for the early flowering phenotype, as well as for the curly leaves and dwarf adult plants [55, 56]. Also the null *clf-50* allele, regardless of the different genetic background (Ws), displayed similar phenotypes, which were enhanced by the *swn-1* weak allele [46]. In addition, it has been shown that *clf-29* display an increase in the number of root meristematic cells [41, 57]), conversely to the *swn-7* allele which has a shorter root with no difference in meristem size [41].

The *clf-28 swn-7* double mutant lacking both EZH2 subunits [41], and the *fertilization independent endosperm 2* (*fie*) mutant, which lacks the Arabidopsis homolog of the DROSOPHILA EXTRA SEX COMBS (ESC) PRC2 subunit [14], have shorter roots and smaller meristems with fewer meristematic cells than wild-type. Consistently, seedlings treated with RDS 3434 displayed a decrease of both root meristem size and meristematic cell number, due to an effect of the inhibitor on the elongation/differentiation potential of meristematic cells.

In Drosophila and in animal stem cells the function of the PRC2 complex is counteracted by the Trithorax Group (TrxG) proteins, which catalyze the trimethylation of lysine 4 of histone 3 (H3K4me3) that acts as a transcriptional activator epigenetic mark [58–61]. In Drosophila and mammals the silencing effect of H3K27me3 is counteracted by the inductive action of H3K4me3 [60, 62]. In Arabidopsis, genome-wide analysis of H3K4me3 and H3K27me3 revealed that only a number of genes are targets of both these antagonistic chromatin marks [37, 38]. Among these genes, key regulators of flower development in the vegetative-to-reproductive transition have been shown to be transcriptionally regulated by H3K4me3 and H3K27me3 [63]. As for the seed-to-seedling transition, a switch from an activated to a repressed state associated to H3K4me3 and H3K27me3, respectively, has been reported for a number of seed developmental genes during germination and early seedling development [26, 33]. However, the antagonism between these two epigenetic marks in Arabidopis is still controversial [64] and it has been recently proposed that Trx proteins cooperate with the PRC2 proteins to repress seed-specific genes during germination and seedling development [65]. In addition, comparative analysis of wild-type and *fie* mutant seedlings lacking a functional PRC2 revealed a genome-wide absence of the H3K27me3 mark in the *fie* mutant; of the H3K27me3 mark-free PRC2 target genes in *fie* seedlings, only a limited number are transcriptionally induced and associated with the H3K4me3 activating mark [14].

In agreement with these results, we show that removal of the repressive mark H3K27me3 involves the establishment of the activating mark H3K4me3 only to a certain degree, since RDS 3434-treated seedlings showed only a slight, although significant, increase in the level of H3K4me3-marked proteins.

Besides being effective in Arabidopsis, we also show that the RDS 3434 inhibitor functions only on the H3K27me3 epigenetic mark; indeed, this compound does not inhibit the methyltransferases of the SET DOMAIN GROUP 8 (SDG8), which catalyzes trimethylation of H3K36 [66], as the entire bulk of H3K36me3 was not significantly different in treated samples compared to the untreated control. In addition, expression of the non-PRC2 target gene *SAUR14* (see below) is unchanged in treated samples compared to the control, thus corroborating the selectivity of this inhibitor.

## Conclusions

Our results indicate that RDS 3434, a compound previously characterised as inhibitor of the human EZH2 subunit, is an effective and selective inhibitor of the PRC2 complex also in plants.

This study can be of significant interest for the community investigating Polycomb activity in plants, as no chemical inhibition specific for H3K27me3 has been reported so far; RDS 3434 could represent a powerful tool to further investigate the effects of the transcriptional control mediated by PRC2 in plants.

## Methods

### General

Reagents and solvents employed were obtained from Aldrich. Melting point (uncorrected) of RDS 3434 was determined in open Pyrex capillary tubes using a Buchi 510 melting point apparatus. NMR spectrum of the synthesized compound was run on a Bruker Avance system, operating at 400 MHz; the solvent used for NMR analysis was DMSO-*d*_6_. Microwave reactions were conducted using a CEM Discover system unit consisting of a continuous focused microwave-power delivery system. The temperature was monitored using a calibrated infrared temperature control mounted under the reaction vessel. The contents of the vessel are stirred by means of a rotating magnetic plate located below the floor of the microwave cavity.

### Synthesis of compound RDS 3434

Derivative RDS 3434 was synthesized by microwave-assisted condensation of propanone with 3-bromo-4-methoxy-benzaldehyde (Fig. 1) following a previously reported synthetic approach [67]. 3-Bromo-4-methoxy-benzaldehyde (1.5 mmol) was dissolved in MeOH and treated with montmorillonite K-10 (600 mg). Evaporation of the solvent gave a dispersion that was suspended in propanone (0.75 mmol) and irradiated at 100 W for 5 min at 100 °C. After cooling, the mixture was diluted with MeOH, filtered and evaporated to dryness. The crude was purified by column chromatography (SiO_2_, chloroform/*n*-hexane 9:1) and recrystallized from MeOH to give the pure product as a yellow solid (51% yield). mp: 154–156 °C; IR *ν* 1554 (C=O ketone) cm^− 1^; ^1^H NMR (DMSO *d*_*6*_, *δ*): 3.90 (s, 6H), 7.12–7.25 (m, 4H), 7.55–7.75 (m, 4H), 8.05 (s, 2H).

### Plant material and growth conditions

The *Arabidopsis thaliana* wild-type line used in this work is Ws-4, unless specified, and was grown in a growth chamber at 24/21 °C with 16/ 8-h day/night cycles and light intensity of 300 μmol/m^− 2^ s^− 1^, as previously described [30]. Seeds were surface sterilized and plated on MS agar (halfstrength MS, 0.8% agar, pH 5.7), unless specified, and stratified at 4 °C for three days in the dark. The *clf-29* mutant line is in Col-0 (SALK_N521003), and was kindly provided by Dr. Miguel de Lucas. The marker lines *RCH1:: GFP* and *CYCLINB1;1*_*pro*_*:CDB-GUS* are in Col-0 ecotype and were previously described [68, 69]. The corresponding wild type line was used for the root analysis. The wild-type lines (Ws-4 and Col-0) have been obtained from the European Arabidopsis Stock Centre (arabidopsis.info). As for the treatment with RDS 3434, wild-type seeds were sown on medium supplied with increasing concentrations of RDS 3434 (30, 60, 120 μM), or with an equal volume of its solvent DMSO (Dimethyl sulfoxide), as control.

### Protein extraction and Immunoblot analysis

Five days-old seedlings, grown in the presence of increasing concentrations of RDS 3434 (30, 60, 120 μM), or with an equal volume of its solvent DMSO as controls, were grinded with liquid nitrogen and dissolved in Chromatin Buffer Extraction (Sucrose 0,4 M; Tris Hcl pH 8 10 mM; β-mercaptoethanol 5 mM; PMSF 0,1 mM; Protease inhibitor cocktail 1X, Sigma-Aldrich P9599). The nuclei were pelleted at 4000 rpm for 20 min, at 4 °C, and dissolved in 800 μl ddH2O. The proteins were precipitated with 150 μl of NaOH/ β-mercaptoethanol (138,7 μl 2 N NaOH and 11,25 μl β-mercaptoethanol) and then with 55% TCA solution, for 15 min in ice. Following centrifugation for 20 min at 14000 rpm, 4 °C, the pellet was dissolved in HU Buffer (Urea 8 M; SDS 5%; TrisHCl pH 6,8200 mM; EDTA 0,1 mM; DTT 100 mM; bromophenol blu) and boiled for 10 min at 65 °C. Proteins were separated on a 12% SDS-polyacrylamide gel (Bio-Rad) and blotted on a PVDF Immobilon-P Transfer membrane (Millipore). Detection of proteins was performed with specific antibodies against H3K27me3 (Millipore #07–449), H3K4me3 (Abcam- ab8580) or H3K36me3 (Abcam-ab9050), and against histone H3 (Biorbyt orb10805) as a loading control. The anti-rabbit IgG conjugated to peroxidase was used as a secondary antibody and the signal was detected with ECL system. The values are the average of two biological replicates (except the immunoblot for H3K27me3 that was performed with three biological replicates), presented with SD values. Significant fold enrichments were analyzed by *t*-test (*P ≤ 0,05).

### Expression analysis

Five days-old seedlings, grown in the presence of increasing concentrations of RDS 3434 (30, 60, 120 μM) or with an equal volume of its solvent DMSO as control, were frozen and grinded with liquid nitrogen. Total RNA was extracted and purified according to Lorrai et al. [70]. RT-qPCR assays were performed according to [31]. Relative expression levels were normalized with the *GAPDH* (At3g26650) reference gene, which is not marked by H3K27me3, and are presented by the ratio of the corresponding mRNA level of the DMSO-treated sample, which was set to 1. The primers used are listed in Table 1. Two independent biological replicates were performed, and one representative experiment is reported with SD values. Significant differences were analyzed by *t*-test (*P ≤ 0.05; **P ≤ 0.01).

**Table 1:** Primers used in this study

Gene name	Forward	Reverse
*DAG1*	TTGTCGAAGGTATTGGACCGA	CCGACTGGGACGTTACGAAG
*DAG1 ChIP*	CGCAACAACAACCAACATTC	GCCGTGTTGTTGGTATTTCC
*WRKY70*	CAGGCCAGTTACGTCAATGGGAAAA	GAAATCGCCGCCACCTCCA
*SAUR14*	GTAGTTCCGGTTTCGTACTTGGACC	CTGCAAGGGATTGTGAGGCCA
*GADPH*	GCTGAGGAAGTCAACGCTGC	CGGACACTAGTGGCTCATCG

### Chromatin Immunoprecipitation (ChIP) assay

ChIP assay was performed according to Lorrai et al. [70]. To analyse the epigenetic profile of the *DAG1* and *WRKY70* loci, chromatin from 5 days-old seedlings, grown in the presence of RDS 3434 120 μM or with an equal volume of its solvent DMSO as control, was immunoprecipitated overnight using antibodies against H3K27me3 (Millipore #07–449), or without antibodies as negative control. After reverse cross-linking, the enriched DNA levels were quantified by qPCR using specific primer sets listed in Table 1. The fold enrichment of a specific region was normalized for the Input fraction, to minimize the background differences among the sample. Two replicates were performed, and one representative experiment is reported with SD values. Significant fold enrichments were analyzed by t-test (*P ≤ 0,05).

### Seed germination assay

All seeds used for germination tests were harvested from mature plants grown at the same time, in the same conditions, and stored for 4–5 weeks in the dark under dry conditions at room temperature. For seed germination assays, triplicate sets of 25 seeds were surface sterilized and plated on agar (0.8%) with increasing concentrations of RDS 3434 (60, 120 μM) or with an equal volume of its solvent DMSO as control. Germination rate was scored at 24, 36, 48 and 120 h after imbibition (HAI). Germination rate was calculated as the ratio of seeds with the radicle protruding from the seed coat on the total number of seeds sown (*n* = 25). The values are the mean of two biological replicates presented with SD values. Significant differences were analyzed by *t*-test (*P ≤ 0.05; **P ≤ 0.01).

### Root meristem size analysis

Root analyses were performed on roots from five days-old seedlings, grown on MS agar supplemented with 0.5% sucrose for 3 days, then transferred to the same medium in the presence of RDS 3434 (240 μM) or with an equal volume of its solvent DMSO as control, for 2 days. For light DIC microscopy, samples were mounted on a media containing chloral hydrate (SigmaAldrich): 3 parts glycerol: 1 parts water. Images were acquired utilizing Nomarski optics under a Zeiss Axio Imager.A2 microscope with a dry 40X objective. For all the analyses, at least 30 samples were analyzed and statistically treated. Root meristem size was measured based on the number of cortex cells in a file extending from the quiescent center to the first elongated cortex cell excluded as previously described [69]. Images were obtained using a confocal laser scanning microscope (Zeiss LSM 780). The length of both the first elongated cell and differentiated cell was measured using image J. Significant differences were analyzed by *t*-test (*P ≤ 0.05; **P ≤ 0.01).

### Statistical analysis

Each experiment was performed in duplicate and repeated with two or three independent biological replicates. Results are expressed as mean (except for the expression analysis) ± standard deviation (SD). Two-tailed Student’s t-test was used to evaluate statistical significance, respect to the DMSO control (*P ≤ 0.05; **P ≤ 0.01).

## Supplementary information

**Additional file 1. Figure S1.** Treatment with RDS 3434 reduces H3K27me3 protein level in the *curly leaf-29* mutant.**a, b** Immunoblot of 5 days-old *clf-29* seedlings directly grown with RDS 3434 (120 μM) or DMSO as control (**a**), and of DMSO- or RDS 3434-treated wild-type (Col) and *clf-29* seedlings (**b**). Total proteins were probed with H3K27me3 specific antibodies, and H3 was used as loading control. Western blot (top) and densitometric analysis (bottom). In (**b**) is shown the relative H3K27me3 protein level (bottom left), and the ratio of DMSO- and 120 μM RDS 3434-treated *clf-29*/WT (bottom right). Results were obtained from two independent replicates with SD values. Significant differences were analyzed by *t*-test (*P ≤ 0.05).

**Additional file 2. Figure S2.** Treatment with RDS3434 of *clf-29* mutant seeds affects seed germination. **a** Seed germination assays of *clf-29* mutant seeds imbibed in the presence of RDS 3434 (120 μM) or DMSO as control. Germination rate was scored at 24, 36, 48 and 120 HAI (Hours After Imbibition). Data represent the mean of two independent biological replicates each performed in duplicate (25 seeds per replica). Significant differences were analyzed by *t*-test (*P ≤ 0.05, **P ≤ 0.01). **b** 5 days-old *clf-29* mutant seedlings directly grown for 5 days in the presence of RDS 3434 (120 μM) or DMSO as control.

**Additional file 3. Figure S3.** Wild-type seedlings treated with 240 μM RDS 3434. 5 days-old wild-type (Ws-4) seedlings directly grown in the presence of of RDS 3434 (240 μM) or DMSO as control.

## Data Availability

The data sets supporting the results of this article are included within the article.

## Abbreviations

CLF: CURLY LEAF
CYCB::GUS: CYCLINB1;1_pro_:CDB-GUS
DAG1: DOF AFFECTING GERMINATION1
DMSO: Dimethyl sulfoxide
DZNep: 3-deazaneplanocin
E(Z): ENHANCER OF ZESTE
EBS: EARLY BOLTING IN SHORT DAYS
EMF: EMBRYONIC FLOWER
EMF1: EMBRYONIC FLOWER 1
ESC: EXTRA SEX COMBS
EZH2: ENHANCER OF ZESTE HOMOLOG 2
fie: Fertilization independent endosperm
FIS: FERTILISATION INDEPENDENT SEED
H3K27me3: Histone H3 at lysine 27 trimethylated
H3K4me3: Histone H3 at lysine 4 trimethylated
HAI: Hours after imbibition
MEA: MEDEA
NMR: Nuclear Magnetic Resonance
NURF55: Nuclear remodeling factor
PcG: Polycomb group
PRC1, PRC2: Polycomb Repressive Complex 1, 2
RCH1: ROOT CLAVATA HOMOLOG1
RDS 3434: 1,5-bis(3-bromo-4-methoxyphenyl)penta-1,4-dien-3-one
RT-qPCR: Reverse transcriptase- quantitative polymerase chain reaction
SAUR14: SMALL AUXIN UP RNA 14
SDG8: SET DOMAIN GROUP 8
SHL: SHORT LIFE
SU(Z)12: SUPPRESSOR OF ZESTE 12
SWN: SWINGER
TrxG: Trithorax Group
VRN: VERNALIZATION
