# Supplementary figures and images for: Inhibition of Polycomb Repressive Complex 2 activity reduces trimethylation of H3K27 and affects development in Arabidopsis seedlings
Source: BMC Plant Biol. 2019 Oct 16;19:429. doi: 10.1186/s12870-019-2057-7 (PMC6796367; doi:10.1186/s12870-019-2057-7)

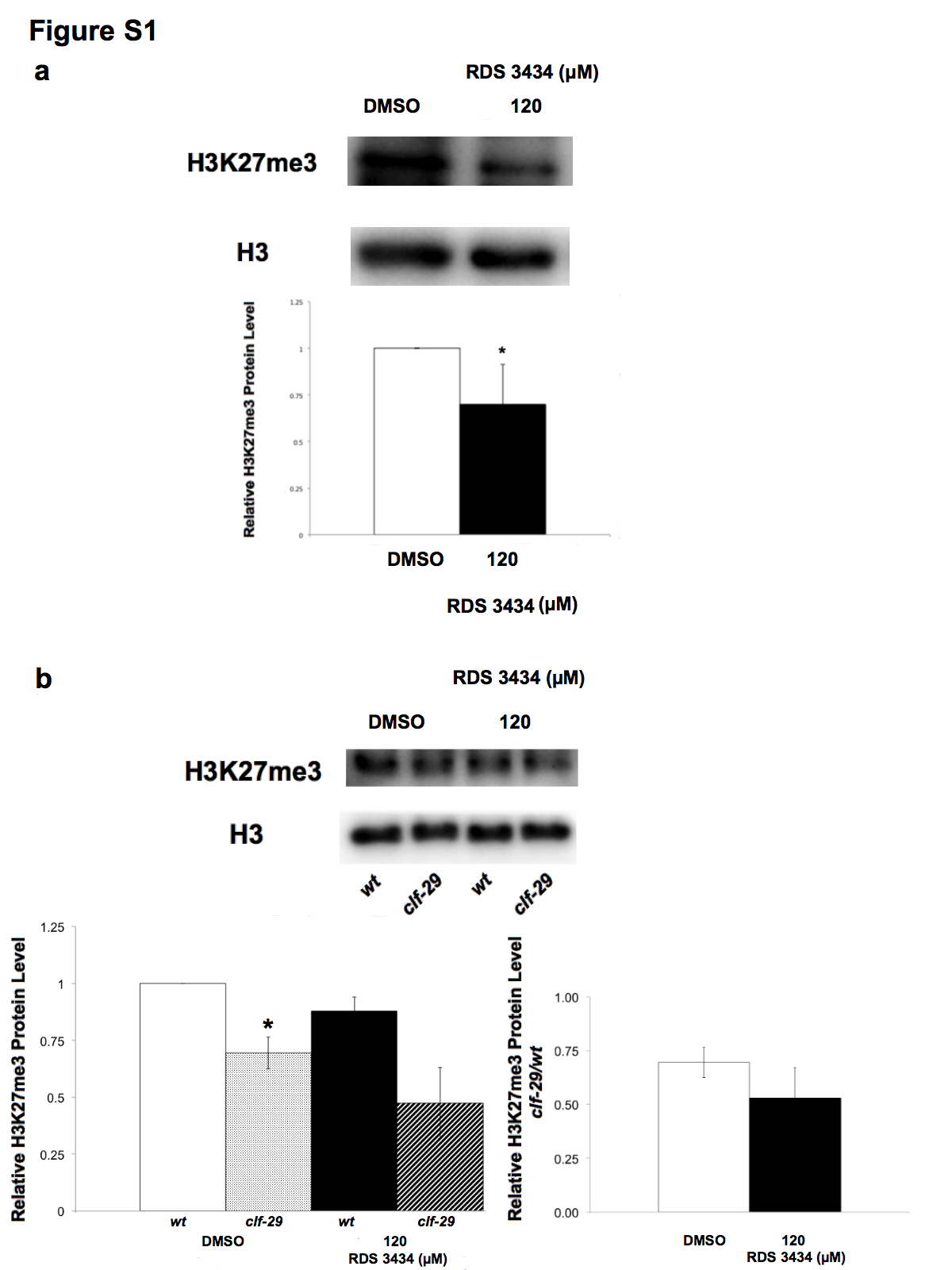

Supplement: Supplementary file 1 — Additional file 1. Figure S1. Treatment with RDS 3434 reduces H3K27me3 protein level in the curly leaf-29 mutant.a, b Immunoblot of 5 days-old clf-29 seedlings directly grown with RDS 3434 (120 μM) or DMSO as control (a), and of DMSO- or RDS 3434-treated wild-type (Col) and clf-29 seedlings (b). Total proteins were probed with H3K27me3 specific antibodies, and H3 was used as loading control. Western blot (top) and densitometric analysis (bottom). In (b) is shown the relative H3K27me3 protein level (bottom left), and the ratio of DMSO- and 120 μM RDS 3434-treated clf-29/WT (bottom right). Results were obtained from two independent replicates with SD values. Significant differences were analyzed by t-test (*P ≤ 0.05). [file 12870_2019_2057_MOESM1_ESM.png]

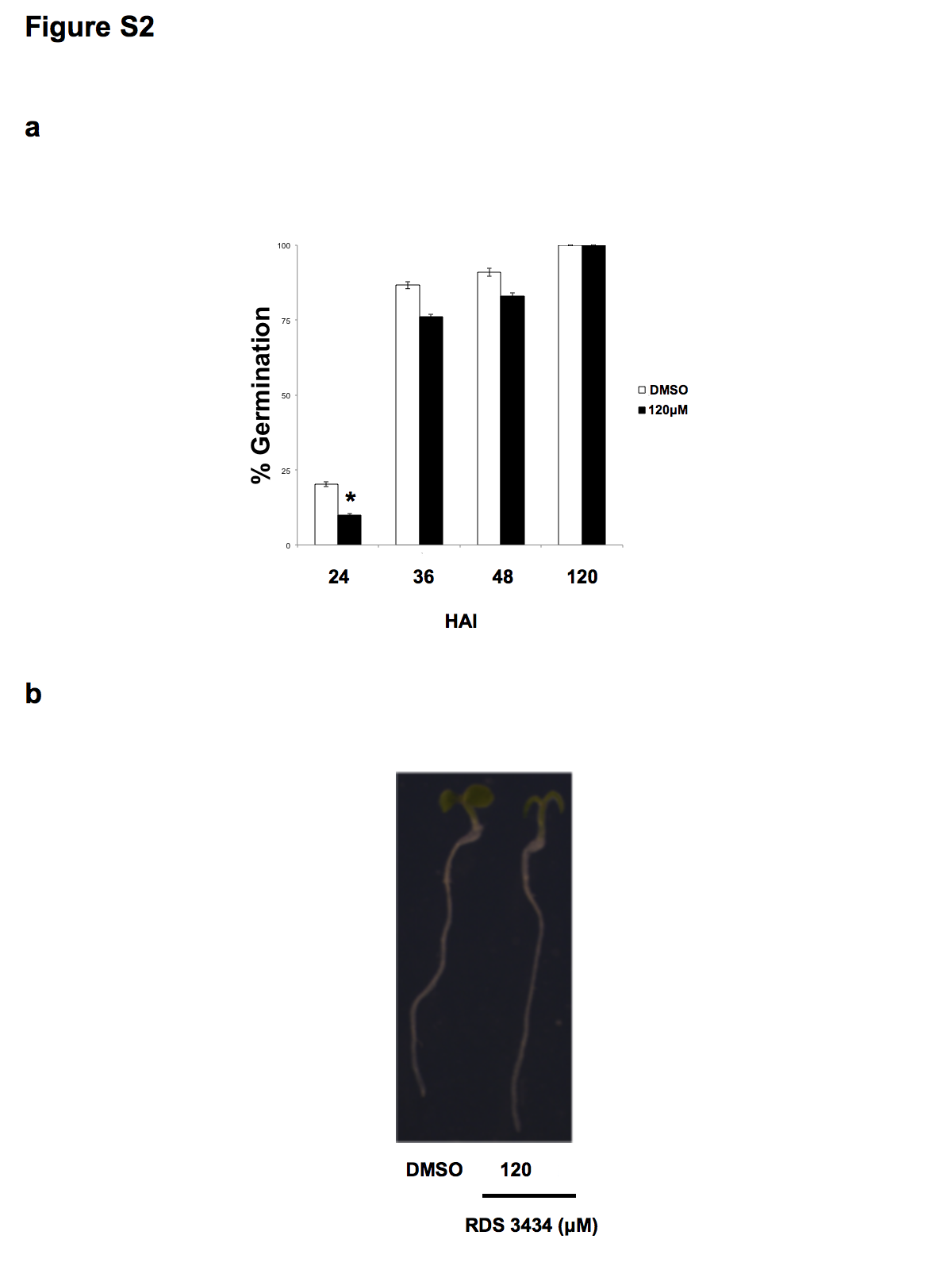

Supplement: Supplementary file 2 — Additional file 2. Figure S2. Treatment with RDS3434 of clf-29 mutant seeds affects seed germination. a Seed germination assays of clf-29 mutant seeds imbibed in the presence of RDS 3434 (120 μM) or DMSO as control. Germination rate was scored at 24, 36, 48 and 120 HAI (Hours After Imbibition). Data represent the mean of two independent biological replicates each performed in duplicate (25 seeds per replica). Significant differences were analyzed by t-test (*P ≤ 0.05, **P ≤ 0.01). b 5 days-old clf-29 mutant seedlings directly grown for 5 days in the presence of RDS 3434 (120 μM) or DMSO as control. [file 12870_2019_2057_MOESM2_ESM.png]

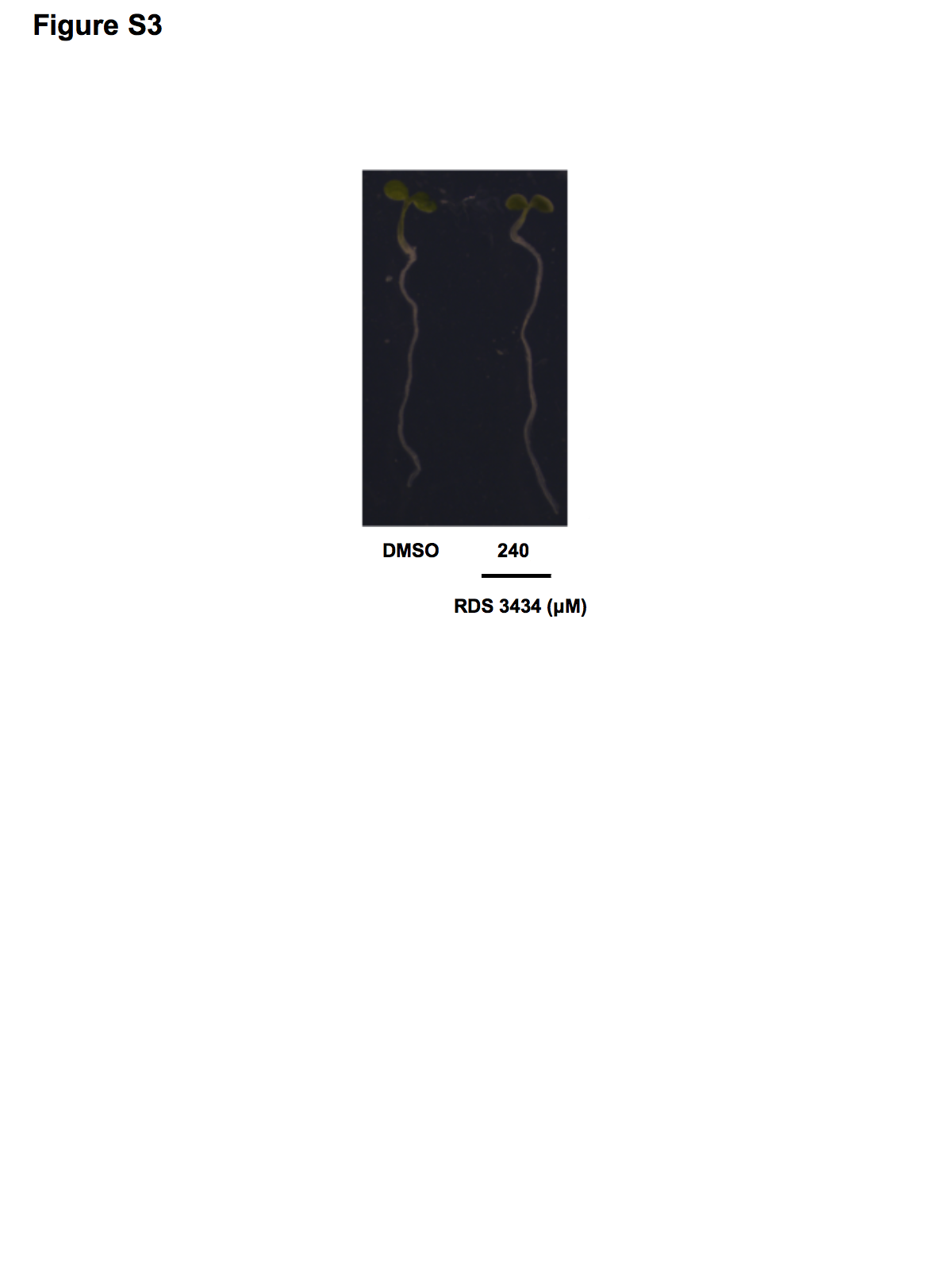

Supplement: Supplementary file 3 — Additional file 3. Figure S3. Wild-type seedlings treated with 240 μM RDS 3434. 5 days-old wild-type (Ws-4) seedlings directly grown in the presence of of RDS 3434 (240 μM) or DMSO as control. [file 12870_2019_2057_MOESM3_ESM.png]
